# The Passions of the Soul
*"The Passions of the Human Soul." By Charles Fourier. Translated by John Reynell Morell. (New issue.) Lea, 1852, London.


**Published:** 1853-04-01

**Authors:** 


// THE JOURNAL

OF

PSYCHOLOGICAL MEDICINE

AND

MENTAL PATHOLOGY.

APRIL 1, 1853.

### Art. I.—THE PASSIONS OF THE SOUL.*

The reason why Socialism has become one of the prominent moral phenomena of our age is, that, with all its extravagances, it sets out from a great and crying want of society. The amelioration of the condition of the masses of mankind, in civilized countries, has become an especial demand, consequent, in great part, on the teeming increase of population. In England and France, particularly, socialism has assumed the character of a political element; and in the latter country, (as though to prove how extremes may meet,) its formidable growth, of late years, has been made one apology at least for the new Bonapartean empire, and the despotism which marks it. The phase of the social system which has held the most sway over the popular mind in France is that of Fourier. It has not been uncommon to confound his theory with the St. Simonian; but it is certain that Fourier had developed his views, in his own mind at least, many years before the doctrines of St. Simon attained to any influence. It is to Fourier that we must attribute the origin of the phalansterian system.

Charles Fourier was born in 1772, at Besançon, and he died at Paris, in his 66th year. His father was a tradesman; and the son, at 18, was placed with a draper at Rouen. From this city he went to Lyons, where he passed the greater part of his life as a merchant. His education was tolerable. He read the classic authors, on the basis of the Latin that he was taught at school; and he was well versed in history

* "The Passions of the Human Soul." By Charles Fourier. Translated by John Reynell Morell. (New issue.) Lea, 1852, London.

and geography. The two latter seem to have been his favourite pursuits, next to his philosophical and social speculations; for to these latter his mind was devoted with the utmost ardour. His character appears to have been genuine and sincere. He first took a disgust to general business from having been reproved for telling a customer the cost price of some article in his father's shop: but he was doomed to be a merchant, for his connexions could not procure for him a commission in the army, which he would greatly have preferred to commercial life. A visit to Paris embittered him against what he termed the "extortions of commerce;" for, unluckily, he was asked four sous for an apple, which he knew he could have bought at the rate of one sou per dozen in his native province. The resistance made by the city of Lyons to the Convention, in 1796, issued in the ruin of our young and reluctant merchant. He now joined the army as a private soldier, but was soon obliged to quit it from ill health. He became clerk in a mercantile house at Marseilles; and here again he was shocked by the monopolies of commerce, and more especially when his employer, during a period of famine, threw a large quantity of hoarded rice into the sea, as it had been kept till it was unfit for use.

These, and other circumstances, fostered the bent of Fourier's mind towards questions of social, political, and commercial progress; and he now began to form his theory of universal unity, from which to deduce methods of practical association. Various articles on these subjects were published in 1803, in one of the Lyons journals; and in 1808, appeared a prospectus of his system, under the title of "La Théorie des Quatre Mouvements." This book, however, its author called in, almost as soon as published. In 1814, Fourier retired to Bellay, in the department of Ain, where he wrote his great work on universal unity, of which the chief part came out in 1822, entitled "L'Association Domestique Agricole," and "La Théorie de l'Unité Universelle." This work mostly consists of the theory and methods of association, but includes also a mass of philosophical and metaphysical speculations. Seven other volumes of manuscript were left at his death, of which some have been published, as the posthumous works of Fourier, in the monthly review called "La Phalange." The work named at the head of this article, "The Passions of the Human Soul," is a translation of one of these posthumous volumes; and it contains also a few extracts from some of the others.

In 1829, our author published his theory of association in a condensed form, under the title of "Le Nouveau Monde Industriel et Societaire." This work brought him into more public notice; but at the moment when the French government were negotiating with him, in respect to an experiment on his social system, his hopes of government patronage for it were blighted by the revolution of 1830, which drove Charles X. from his throne.

After his death Fourier's school grew rapidly, and his schemes were maintained by a daily paper entitled "La Democratie Pacifique," which ceased after the revolution of 1848, when Louis Philippe's reign ended, and gave place to the Republic, under which its principal editors, too republican for this *nominis umbra*, were banished. In this revolution, "La Phalange" also fell, and full half of Fourier's works have never seen the light. Mr. Hugh Doherty, who has prefixed to "the Passions" some critical annotations etc., became personally acquainted with Fourier, in Paris, where he lived on 60*l.* a-year, and left 40*l.* of ready money in his cash-box, at his death. Mr. Doherty adds :—"His conversation was sometimes animated and witty; but his general bearing was slightly tinged with melancholy, and indifference to current notions and opinions. He never married." An ample biography of him may be found in Pellarin's "Vie de Fourier," of which an English translation has been lately published in America; and in Mr. Doherty's article, entitled "Fourier," in the "Penny Cyclopædia."

The writings of Fourier, embrace a very wide field of inquiry. He not only directed his attention to social and political economy and commerce, but also to cosmogony, psychology, the history of philosophy, and ethics. He has treated all these subjects in a manner, and with a phraseology peculiar to himself, and has regarded them as parts of one general system of nature, united by one principle, or universal law, which he terms, the *law of movement.* His theory of the passions has been published in the "Phalange," since his death, and the importance of this theory to his views in general, is evident from the fact that he founds his entire system on his analysis of this part of human nature.

According to Fourier, reason is the grand instrument by which man acquires truth. He gains this gradually, and after many failures; and the social system will be only tentative until "*universal harmony*" is attained by the true laws of nature being discovered. Man is God's image, and this, though now distorted, admits of rectification by a true social theory. Man, therefore, is mainly to be studied—in his organization, his intellect, his soul, and in his history. The latter exhibits man as passing through various stages, like those of the individuals of his species. There is the infancy of society, its childhood, its adolescence, its manhood, its old age. The principle of unity is always advancing, though but slowly, in these stages; but it will at last be attained in perfection. The grand vice of society has always been *individualism,* or private selfishness. This must give place to the public good; and then individual and general happiness will blend, in the state of

*harmony.* Man's body is only the organ of his higher nature: his intellect is his regulative principle: his passions are his sole active impulsive force. We must look to the *will,* and to the passions which influence it, in order to find the real man.

The passions and attractions of the soul are the springs which lead to all action. Fourier classes them as follows: The wants of the body are tendencies to physical enjoyments. They are seen in the *five senses,* which are five special passions or attractions, causing man to act in various ways for the purpose of satisfying them. Also, the *affections of the soul itself* produce action. These affections may be resolved into those of *sociability and sympathy,* by which man is impelled, in various circumstances, to seek the fellowship of man. The principal varieties of affection which cause men to congregate in different forms and groups, are the feelings of friendship, love, family affection, and corporate association: he calls the two latter *familism* and *ambition.*

There are, also, according to Fourier, in addition to these special wants of the body and the soul, certain more general wants which are incorporated intimately with man's inward life. He terms these,—wants of *unity* and *order;* the first being, as he says, the climax of all others in man's natural and spiritual wants, and the latter being a neutral or mixed class of wants, consisting of the love of variety or alteration, the love of refinement (or intrigue) and emulation, and the love of combination or cumulative action. These wants are classed by Fourier after the manner of the *gamut* in music, with which, according to his notions, they had a strong analogy; the elementary forces and attractions of feeling and passion having, as he thought, certain relations corresponding very much with those of the musical octave of elementary notes. This analogy, which, we dare say, strictly carried out, will appear to most of our readers, as it surely is, fanciful enough, will strikingly exemplify the character of Fourier's mind. We cannot do better than give the table of these analogies, which we take as it stands in the prefixed critical annotations and general introduction of Mr. Doherty, to whom we must express our obligations for the assistance he has afforded, in enabling us to condense into a compendious form the extended dissertations of Fourier, reaching to an amount of nearly 900 pages. Indeed the English public are by no means less indebted to Mr. Doherty for the share he has had in the publication of these two volumes (in one), than they are to the translator himself, for having made vernacular a system which, whatever be its merits in other respects, at least contains analyses of the "wants of the soul" which are extraordinary examples of the elaboration and ingenuity of the author.

The following is Fourier's scale of the wants of man's body, and of the feelings and affections of his soul. Our readers will take it as a curiosity.

| | *Scale of the Passions.* | *Scale of Musical Notes.* |
| --- | --- | --- |
| Sensuous passions or attractions . . . . | 1. Sight. | ♯ 1st, half-tone, flat or sharp. |
| | 2. Hearing. | ♯ 2nd. ,, ,, |
| | 3. Taste. | ♯ 3rd, ,, ,, |
| | 4. Smell. | ♯ 4th, ,, ,, |
| | 5. Touch. | ♯ 5th, ,, ,, |
| Affections . . . . . | 6. Friendship. | DO, or tonic note. |
| | 7. Love. | MI, or mediant note. |
| | 8. Familism. | SOL, or dominant note. |
| | 9. Ambition. | SI, or sensitive note. |
| Distributive passions, or the love of order . . | 10. Emulation. | RE, or sub-mediant note. |
| | 11. Alternation. | FA, or sub-dominant note. |
| | 12. Cumulation. | LA, or tonic of the minor key. |
| | ⋈ UNITYISM. | DO, unison, or octave note. |

Our readers, even those of them who know the theory of music, will probably be surprised or amused to learn that, in this scale, and in his "laws" and "parallels" of "elementary forces," Fourier's imaginative and inventive genius clearly saw principles which were complete in all their bearings, and which readily accounted for all the actions, passions, and impulses of the life of man. Our psychological readers will perhaps have suggested to their minds, here, the theory of Descartes—that all our ideas which are perfectly distinct and clear to our own consciousness are true; and they will at the same time, perhaps, think, that Fourier's table (at least in its musical part), is a good illustration of the total untenableness of that doctrine, so far as it refers to *objective* truth; and will regard it as a proof that Descartes' principle required the correction which it received from the hand of Leibnitz, in his principles of "contradiction" and the "sufficient reason." Surely, even in sane minds, we have not far to go in order to refute a great fundamental error of one who was, notwithstanding, co-ordinately with Bacon, the joint-patriarch of modern philosophical speculation: for how "clearly," often, do men see what they wish to be true!

Fourier held that while the motive springs of life in the animals below man were inferior in degree and power, they were, nevertheless, exactly similar in nature. The life and growth of plants is analogous to those of man in organism and function, though not of the same nature in life and essence. But Fourier carried his principle of unity further than this. Diogenes of Appollonia made the atmosphere a living intelligence, and the whole universe an animated being spontaneously evolving itself: he attributed to the world a set of respiratory organs, which he saw in the motions of the stars:—but what will be thought of a philosopher of our own times who was so enamoured of his theory of man as to apply it to the heavenly bodies? Fourier actually believed the *planets* to be living beings, superior to man, but still endowed with the same passions and attractions; and that it was these that impelled them to associate in groups and solar systems, just as human beings congregate together in society! Our author, like all men who are in love with one idea, was not content with any moderate application of it—the one idea must reign throughout the universe. Fourier asserted that the same attractions and impulsions which cause man to act, and which produce life and motion in all animated beings, (whether men or *planets!*) also impel the Deity, and cause Him to act in the creation:—this Fourier thought he proved from scripture. To his sanguine and imaginative mind, the laws of the whole universe of being were now discovered; the key was found for unlocking all the arcana of nature: the mysteries of life and motion, of phenomena and noumena, of time and space, of the natural and the spiritual, of the visible and the invisible, of causes and effects, were all unravelled. Hence we have the "theory of universal movement or phenomenal effects—of universal attraction or impulsive causes—and of universal analogy or correspondency: and these three together constitute his "*theory of universal unity.*" The theory of movement is derived from the analysis of human life, that of attraction from the analysis of human wants and feelings, and that of analogy from the analysis of that manifest parallelism which exists between the impulses of one animated being and those of another.

From all these analyses, which embrace all the life and motion in the universe, Fourier deduced the following four *axioms*: the law of series rules the distribution of the harmonies of nature; attractions are proportional to destinies; analogy is a universal law; there is unity of system in nature. By means of these elements of universal method, our author attacks the most difficult problems of philosophy, of history, and of sociology. In his "analysis of universals," he defines the "first principles of nature," as 1, the active principle or *spirit;* 2, the passive principle or *matter;* 3, the neuter principle or *mathematics.* These principles, Fourier says, are analogous, respectively, to the senses, the affections, and the distributive passions of man.

We cannot refrain from a brief quotation in reference to Fourier's views on Cosmogony, an ominous term, in itself, but one which, to geniuses like our philosopher, only stood for a region of familiar truth, and by no means a *terra incognita.*

"The planets procreate their own species: the elephant, the oak, and the diamond, were created by the Sun; the horse, the lily, and the ruby, by Saturn; the cow, jonquil, and topaz, by Jupiter; the dog, the violet, and the opal, by our Earth; all the moons and planets have created special series, classes, orders, and varieties of animals, vegetables, and minerals upon our globe, and also on each moon and planet of our solar system."

We must not, however, be led away from his theory of human nature to his cosmogony. Mr. Doherty may well say here: "Fourier's observations and analogies on these subjects are exceedingly ingenious, though devoid of positive inductive logic and philosophy."

Fourier maintains that social life exists in two different states, analogous to those of life in the womb and after birth. We are at present living in the womb of darkness, society being a partially developed fœtus, or a sort of caterpillar which crawls upon the earth before it is transformed into a butterfly. This condition of society admits of various degrees of progress and development, which Fourier enumerates by the names of Edenism, savageism, patriarchism, barbarism, civilization, guaranteeism, socialism, and harmonism. Our author's phalansterian views are based on what he terms the *phalanx,* or industrial hive, which is the social unit of his system. A thousand individuals, or more, he tells us, are necessary to form this perfect social or industrial hive, having within itself the means of feeding, clothing, lodging, educating, and governing all its members, in a permanent, complete, and satisfactory manner; but as many as about 1600 persons, of all ages and both sexes, are required to form a completely self-supporting social community. For the performance of the necessary work of an association, 810 healthy and active individuals are required, says Fourier, and twice that number would be necessary, in order to insure the constant activity of 1000 persons. He divides this social body of 1600 people into sixteen *tribes.* The first tribe is that of infants under 4 years; the second, that of children from 4 to 7; the last tribe being that of declining age, from 70 to the end of life. Youth, adolescence, maturity, and declining age, are each subdivided into cycles of about five years, so that the whole sixteen tribes are formed of different ages from infancy to second childhood. Each tribe again contains two *choirs,* male and female. The thirty-two choirs form a *vortex,* or social and industrial self-supporting hive, *phalanx,* or associative unity. The head of a phalanx is termed a *monarch,* or governor of a single community. The ruler of a union of phalanges is termed a *duarch.* The social hierarchy goes on to triarchs, tetrarchs, pentarchs, etc.; to the *douzarch,* who governs a whole continent; while the *omniarch* rules the entire globe. He is, in fact, as it were, the civil pope of the whole social system; but amidst all these gradations of power, the people who originate them are supreme.

Whether any part of this phalansterianism shall ever be found practicable in society or not, there are on the face of it traces of the same extravagance and fancy which characterise the greater part of all Fourier's theoretic speculations. The notion of a cosmical omniarch stands much on a par with his representation of universal matter by the number 5, universal spirit by the number 4, universal intellect by the number 3, and their "trinity in unity" by 12, his sacred number for all the perfect harmony in nature. Nevertheless his writings constitute a vast mass of most acute and elaborate speculation on the social science, which all future philanthropists will peruse with intense interest, and from which they will draw many valuable suggestions for more sober and practical methods; for no one could have written as Fourier did, without having most closely and profoundly studied human nature.

The volume before us is chiefly limited to an analysis of the "soul," as distinguished from the body on the one hand, and the intellect on the other: here, by *soul*, it is evident that Fourier understands the affections, emotions, and passions. We have derived much instruction, in connexion with our perusal of the work, from Mr. Doherty's criticisms of it. We agree with him, that Fourier's analysis of the passions and attractions of the soul is "incomplete;" but we are far more impressed with the fanciful and affected character of the whole work than his candid critic appears to be. The strangeness and barbarism of the terms, the jargon which they must present to every ordinary reader, are repulsive in the extreme. The thoughts, moreover, are often, very often, to us at least, as strange and confused as the terms in which they are expressed; but we must hear the candid testimony of Mr. Doherty himself:

"I was previously a diligent and somewhat zealous student of Fourier's system. Many parts of it appeared to me sublime, while others seemed more plausible than rational. There was, however, powerful originality, and truth enough in the whole thing to merit the most conscientious study. This I undertook, and carried on for years, without being satisfied. Fourier I conceived consistent with himself in most things, but not invariably. I found it difficult, however, to refute his propositions as he states them, and yet I could not feel sympathy with his most startling views and theories.—(*Introduction*, p. 36.)

We quite agree with our critic that it is desirable "to put the reader on his guard against Fourier's notions of morality, which mingle with the regular analysis of sentiments and passions in the present work." Whatever truth, moreover, there may be in the facts of Fourier, adduced or assumed, in his analysis of elementary tones or attractions in the soul, we accord with the remark, that these facts, "in their relative connexions and arrangements are imaginary." We will quote, in a brief form, a few other passages from Mr. Doherty which express much our ideas of some of Fourier's details:

"The wants of the body are not confined to those of the five senses, nor are the wants of the soul confined to those of the four affections, and the three distributives described in his analysis. The subversive and harmonic developments of passion, too, as Fourier describes them, are not always correct. Subversion is confounded with perversion. Selfishness is not necessarily the social root of evil, nor is unityism always the root of good. Self love and social love may produce good and evil actions, as circumstances may determine. A man may do good from selfish motives, and work mischief from the purest love. The passions are no doubt subject to various modes of development in different states of progress and refinement, but morbid feelings and desires are accidental, and not essential parts of nature, like roots of trees. The passions of the soul, in fact, cannot be logically classed as a ramified tree."

"According to his first division, the five senses tend exclusively to *luxism*, or voluptuousness; the four affections to *groupism*, or sociability; the three distributives to *seriism*, or *social order*. This is a mistake at the very root of his analysis. The wants of the body are not strictly confined to those he mentioned; the three distributives, as Fourier himself perceived in his anticipations of objections, are not special wants at all, but general wants of the whole body, soul, and mind, in their collective progress and development."

"The sober method of inductive science cannot build upon imaginary theories of number and analogy without due observation and experience. The elements of music in the gamut are of twelve degrees or tones, and a full set of human teeth are thirty-two in number; but undoubted facts are no sufficient warrant, even in analogy, for us to guess that human passions and attractions are distributed in the same numbers and varieties. Preconceived ideas of numbers, scales, and formulæ, are delusive snares of method, which imprison the imagination in a vicious circle of analogy in every branch of study and investigation. I must here observe, however, that Fourier's analysis of the three distributive wants of the soul, in unison with general progression, is a masterpiece of ingenuity, and none the less from being wrongly-classed in his imaginary scale of twelve."

In our perusal of Fourier's work, nothing has more struck us than the absence of that close psychological analysis which has distinguished the great metaphysicians of different schools and systems. For it should be remembered, that the author aimed not only at a social theory, but at a system of philosophy in general. With justice, therefore, as appears to us, does Mr. Doherty remark: "The grand defect of Fourier's theory, as a theory of the passions and attractions, lies in the total absence of mental analysis." "This," adds his critic, "has been generally felt by his disciples; but they are not able to correct the error. They have classed the three *distributives* as mental passions, and supposed the problem solved. The difficulty was thus set aside, not overcome."

"Fourier's intuitive idea of unity in all the harmonies of nature was correct, but his observations and analysis were incomplete. In his system there are multitudes of useless and erroneous complications, arising from mistaken views of unity and variety. . . . . The most remarkable part of his analysis is that of the ruling passions of the soul, which form the special characters of individuals and groups. Nothing of the sort was ever before systematically attempted by psychologists. It is exceedingly ingenious and instructive, though the numbers he establishes are quite imaginary, and the morals he proclaims are more than doubtful. . . . . He has put us in the way of analyzing characters and ruling passions, but he is as far from having solved the real problem of passional attraction and association, as Copernicus was from the discovery of the laws of planetary gravitation. . . . . . The moral and physical existence of the race must be improved, to some extent, as well as science and mechanical invention, before the highest order of associative unity and harmony can be fully conceived in theory, much less organized in practice. There is a natural growth of society as well as of individual life; social institutions will progress as industry and science are advanced, just as the body and the mind of man progress from infancy to manhood in the individual."

"Fourier's system of association, though imperfect, is worth studying with attention. Its practical suggestions are most valuable. His criticisms on the present state of things are luminous beyond description; his views on the philosophy of history are excellent. Many of the papers published in his posthumous works are indescribably beautiful in thought and inspiration."

We will now give some extracts from Fourier himself, by which our readers will judge of his style of writing, and the great peculiarity of his mode of thinking. We will, however, first premise a few further remarks from Mr. Doherty, who from his long-continued and laborious, we may add, candid and dispassionate study of all Fourier's published writings, is perhaps better entitled to be heard on this subject than almost any other critic. We the more gladly avail ourselves of his remarks on Fourier's style, because we do not happen to have the French original before us, so that our acquaintance with our author is entirely through the medium of the present translation.

"Fourier's style is more original and graphic than pure and elegant. It is in fact quite ungrammatical in many instances, especially in his posthumous writings, which had not been finally corrected for the press. I have read in manuscript the second volume of the translation, and appended some few notes. I can vouch for its fidelity to the original text. When Fourier has created new words, unknown to the French language, it is most difficult to render them in English, not to say impossible."

Mr. Doherty then gives some examples of the negligence of Fourier's mode of writing in the French original. In some cases, words are used which have no strict grammatical connexion with the rest of the sentence, and we are left to guess what Fourier meant to say. Sometimes he appears to mean almost the contrary of what he has verbally written, for what he does say is in contradiction to his theory. These defects of style have been faithfully copied, for the sake of presenting Fourier himself to the public in the translation itself; for Mr. Reynell Morell did not consider himself at liberty to alter the text, whatever might be the grammatical irregularities, which may be said, indeed, to some extent, to be idiomatical in the author. Nevertheless, from our own perusal of the work we can say, that the number of cases in which the apparent meaning of Fourier is obscured from the above cause is not very considerable. Where there is obscurity, it most generally arises from the strangeness of the thoughts, and the fancifulness of the analogies.

We must now content ourselves with a few extracts from the work, which will serve to give our readers some idea of Fourier's method of analysis; these extracts, however, we must forewarn them, are necessarily but partial developments of discussions which are too widely extended for our pages:

"We see a fundamental division in the material universe, which presents us with the harmonic worlds, or planets, and with the subversive worlds, or comets, and with gradations of ranks between the heavenly bodies. We ought to admit the same division in every classification of the passions. To become initiated in the alphabet of their science, you must first study their distribution. They are not of an indeterminate quantity, like the branches of a tree; they are a fixed, and very fixed, number in all their gradations. I here give their table only carried out to the fifth degree:—

| *Pivot Classes.* | *Orders.* | *Genera.* | *Species.* | *Varieties.* |
|---|---|---|---|---|
| Trunk 1 | 2 | 3 | 4 | 5 |
| 1 3 } 4 | 12 } 13 | 32 } 33 | 134 } 135 | 404 } 405 |
| Root 1 } | 1 } | 1 } | 1 } | 1 } |

That is to say, if we examine the trunk of a tree, there is but one passion which is called,

" *Unityism* in harmonic development, or trunk.

" *Egoism* in subversive development, or trunk-root.

"Then, in the first, second, and third degrees, you find the numbers 3, 12, 32, &c., which I change into 4, 13, 33, because in the theory of movement the pivot enters into all the divisions, in the same way that, in the mechanism of the juices of a tree, the trunk communicates with all the branches, and the trunk-root with all the roots."

We cannot pursue this extraordinary theory of the passions; and if we did, we shrewdly guess that Fourier's explanation of it (pp. 5, 6 *seq.*)

would leave the matter to our readers, as it has to us, about as dark as before.

We have not room to give a table which follows, (p. 18,) entitled "Potential Gamut of the Accords of Friendship, and of the Accords of Love, with Analogies." We cannot pursue the author through his Greek compounds: "Heterophily, Monophily, Hemiphily, Multiphily, Phanerophily, Ultraphily, Omniphily, Extraphily," &c., &c.: nor through his "Visuism," under which head we have the "Converging, Asinine, Cameleonic, Co-terrestrial, Co-aerial, Co-aromal, Co-aquatic, Noctambulic, Diaphanic or Co-igneous, and Ultra-ethereal *eye;*" nor can we trace the "Heteromodal, Monomodal, Dimodal, Tetramodal, &c., general accords," which are all given with the gamutic terms *Ut, Re, Mi, Sol, La,* &c., with sharps and flats. In this chapter, however, we have the following intelligible passage, illustrative of the author's assertion, that not only men, but almost all nations fall into an "impotence of some particular sense, in every degree."

"To this class belong the French; without excepting the fashionable world of Paris, which has an exceedingly untrue ear. The French are physical idiots (cretins) in the sense of *hearing.* The following is a proof of it:—I once attended a ball, in Paris, in the Hotel de Marbœuf, at the Champs Elysées, where there was amongst the six musicians a clarinet which was, if not a semitone, at least a quarter of a tone higher than the violins. I pointed it out to two of the stewards of the ball, and asked them if they did not mean to stop that infernal clarionet. One of them showed an utter indifference about the matter; the other said, 'It is true, the instrument is out of tune, but it will do; nobody notices it.' And yet this ball only contained the higher classes—the cream of the ineffables. If the same orchestra had been given to Italian cobblers, they would have hissed, and turned out the criminal. There are, then, it seems, whole nations which are injured, and, as it were, crippled in one of the senses. The French nation is one of these. In the regimental bands you hear two or three instruments in discord, without any body being moved by it, without the musicians or the corps of officers who pay the band appearing to notice it. The populace in France listens eagerly to ballad singers, who are so out of time and tune, that a man who has got an ear is obliged to run away. These auricular butchers are the luxury of the French nation. It is, in music, what the crows are in gastronomy, which only live on putrid food.'

We will now entertain our readers with a final extract, from the chapter on the "*Papillon,* or Love of Alternation," one of the "radical passions"—termed the *papillon* (butterfly) for a reason which at once appears. The passage occurs in the third chapter of the second volume:

"Those who weigh words and not things will think that the *papillon* is the passion of flighty heads: they will consider this name as synonymous with inconstancy and frivolity.

"It is nothing of the kind. The gravest characters are often those that have the papillon among their dominants.

"It is true that the man who had the papillon as his exclusive dominant—a monogyne of papillon, (we call *monogyne* in the scale of characters the man who has only one dominant,) would be a frivolous, inconsequent being, and of little worth; but a character that amalgamates the papillon with several other dominants, such as ambition, friendship, familism,—the cabalist—becomes a man of great resources; and to prove this, Julius Cæsar, the most perfect, the best organized head that was ever seen on the political stage, had not only the papillon among his co-dominants, but he had it as his super-dominant. I give this name to that one of the dominants which has the casting vote, and takes the lead of the others in a character.

"Every one must have seen some of these men who love to carry on at once a crowd of functions, whether of genus or of species; if they are at work in an action of law, they will want to compose four briefs at once, for four different causes. This mania of cumulation reigns even in their recreations. If they are reading a book, they will not finish it unless they have three or four to read at a time—to-day one, to-morrow another. They have a *ricochet** or rebounding memory; it is stronger than memories laboriously cultivated.

"A limited mind, a character of middling title, like the monogynes, (who are the lowest title in the ground scale of characters,) will be apt to think that this multiplicity of enterprises will interfere with the success of each; and that the barrister who labours on four briefs at once—this morning at one, in the evening at another, and to-morrow at a third, will only make a mess of all four. On the contrary, if this barrister is a character co-dominated by the papillon, his four briefs undertaken together will be much better, more complete, better written, than if he had composed them separately, and each of them at one stroke. All the papillonists require functions, broken, cumulated, and dove-tailed. This passion follows, in fact, the course of the pretty insect that represents it, and whose flight is broken or alternate.

"Cæsar dictated at once four letters to his secretaries, and with his own hand he wrote a fifth. Here is a very papillonized imagination; and none was ever seen stronger than Cæsar's.

"In general, the polygyne papillonists have gigantic memories. The epithet of polygyne signifies that the individual has several dominants. If he had the papillon alone, he would be a monogyne of papillon; but if he has other passions as dominants, he is a polygyne of papillon.

"Nature, that distributes faculties according to the uses that she premeditates, has been obliged to give a very strong memory to the papillonists, because they are destined to cumulate many studies or labours. They must be able to embrace with facility five or six times more than a common memory.

* A term in gunnery used for the curves described by mortar firing, as distinguished from the point-blank aim.

"Hence it comes that a papillonist does not retain writings little weighted with matter, and retains easily those that bristle with difficulties. I could retain by once reading twenty German or Arabic names, and I should not recollect four French ones.

"A papillonist will retain from the outset the syllabic chronograms, such as that one which unites all the œcumenical councils in a hexameter verse:

| | | | | | |
|---|---|---|---|---|---|
| Ni-co-e. | Ca-co-co. | Ni-co-la. | La-la-la. | Lu-lu-vi. | Flo-tri. |

That is to say,

| | | | | | |
|---|---|---|---|---|---|
| Nicomedia. | Chalcedon. | Nicomedia. | Lateran. | Lugdunum. | Florence. |
| Constantinople. | Constantinople. | Constantinople. | Lateran. | Lugdunum. | Trident. |
| Ephesus. | Constantinople. | Lateran. | Lateran. | Vienna. | |

"The individual whom I heard recite it, wishing to regale us with a second syllabic chronogram, recited twice this one,

Ba ca da fa, ga la ma na, A B C D, fe ge le me.

"His memory failed when he had to explain this second chronogram, and he declared he no longer knew what it meant.

"A year after this, I recited his two chronograms to him; and I asked him if he recollected the details of the second that he had not been able to explain to us at the time. He was amazed at my remembering exactly his chronograms which he had only twice recited, and whereof I had not taken a note.

"Such is the property of the papillonic memories, to which a heavy load is but a slight burden; they will not retain easy matters, such as French names. If I am given the address of a name very easy to retain, like John or Giles, I shall not remember it the next day; but if barbarous names are pointed out to me, such as Bischoffaverser, Klinkostrom, Oracyewski, Altenkirkhoff, I shall retain twenty by simply reading them, when I should not retain two French names.

"The papillonists are beings that must be overloaded with functions; they are commonly more intelligent in cumulating twenty employments than another man would be in cumulating two. A journalist complained lately, because a certain member of the Institute cumulated twenty-five different functions in his one person. It is possible that he may have performed the twenty-five better than two or three."

From this it is evident that Fourier regarded himself as a "*papillonist,*" at all events; and if our readers shall peruse these two volumes, we warrant them that before they have done, whatever they may think of themselves, they will pronounce the author not less than a first-rate "*polygyne papillonist.*" We may add, that if they are impeded in their comprehension of his doctrines by the terms which he has borrowed from musical science to illustrate them, a complete explanation of these terms will be found in the second section of the first chapter of the treatise "On the Scale of Characters," in the second volume; and in the second section of the second chapter of the first volume, "On the Passional Dominants and Tonics." We close by remarking that much, very much of human nature may be learned by these extraordinary dissertations, and in this consists their sole value; for much, and very much, of warning may also be derived, for the instruction of those who desire, as Fourier did, to benefit mankind—not to allow their theories to be disguised in fanciful analogies, or to run wild in a jargon unintelligible to all but the learned few, if always even to them.

---

